# Automating Electronic Clinical Data Capture for Quality Improvement and Research: The CERTAIN Validation Project of Real World Evidence

**DOI:** 10.5334/egems.211

**Published:** 2018-05-22

**Authors:** Emily Beth Devine, Erik Van Eaton, Megan E. Zadworny, Rebecca Symons, Allison Devlin, David Yanez, Meliha Yetisgen, Katelyn R. Keyloun, Daniel Capurro, Rafael Alfonso-Cristancho, David R. Flum, Peter Tarczy-Hornoch

**Affiliations:** 1 University of Washington, US; 2 Oregon Health and Sciences University, US

**Keywords:** electronic health records, validation studies, comparative effectiveness research, quality improvement

## Abstract

**Background:**

The availability of high fidelity electronic health record (EHR) data is a hallmark of the learning health care system. Washington State’s Surgical Care Outcomes and Assessment Program (SCOAP) is a network of hospitals participating in quality improvement (QI) registries wherein data are manually abstracted from EHRs. To create the Comparative Effectiveness Research and Translation Network (CERTAIN), we semi-automated SCOAP data abstraction using a centralized federated data model, created a central data repository (CDR), and assessed whether these data could be used as real world evidence for QI and research.

**Objectives:**

Describe the validation processes and complexities involved and lessons learned.

**Methods:**

Investigators installed a commercial CDR to retrieve and store data from disparate EHRs. Manual and automated abstraction systems were conducted in parallel (10/2012-7/2013) and validated in three phases using the EHR as the gold standard: 1) ingestion, 2) standardization, and 3) concordance of automated versus manually abstracted cases. Information retrieval statistics were calculated.

**Results:**

Four unaffiliated health systems provided data. Between 6 and 15 percent of data elements were abstracted: 51 to 86 percent from structured data; the remainder using natural language processing (NLP). In phase 1, data ingestion from 12 out of 20 feeds reached 95 percent accuracy. In phase 2, 55 percent of structured data elements performed with 96 to 100 percent accuracy; NLP with 89 to 91 percent accuracy. In phase 3, concordance ranged from 69 to 89 percent. Information retrieval statistics were consistently above 90 percent.

**Conclusions:**

Semi-automated data abstraction may be useful, although raw data collected as a byproduct of health care delivery is not immediately available for use as real world evidence. New approaches to gathering and analyzing extant data are required.

## Introduction

Learning health care systems seek to deliver appropriate and effective health care by leveraging existing health care data [1,2]. These systems repurpose the data entered into electronic health records (EHRs) during the delivery of care for secondary uses, such as quality improvement (QI) initiatives [3]. Through use of tools such as natural language processing (NLP) algorithms that facilitate data abstraction, and creation of central clinical data repositories (CDRs) to aggregate data from multiple nonaffiliated institutions, platforms can be created to facilitate rapid learning. Many national initiatives are well underway. These include the Food and Drug Administration (FDA) Sentinel Initiative [4], Observational Health Data Sciences and Informatics (OHDSI) Program [5], and the National Patient-Centered Clinical Research Network (PCORnet) [6]. Each of these initiatives employs a distributed federated model wherein each participating site standardizes and normalizes their data to network standards, and then forwards those data to the central CDR [7]. These initiatives constitute real world evidence, that is, information on health care derived from multiple sources outside of typical research settings, including information from EHRs, registries, claims data, and wearables [8]. The National Academy of Medicine (formerly Institute of Medicine) has recently published the findings of their workshop on Real World Evidence [9].

The uncertain quality of real world evidence and electronic data has been an ongoing challenge and an area of investigation for more than twenty years. Early efforts focused on business applications [10,11,12]. Wang and Strong, for example, emphasized that the concept of data quality includes contextual quality (relevance, timeliness, completeness), representational quality (concise and consistent representation), and accessibility (ease of access, security). More recently, investigators have applied these concepts to clinical health care data [7,13,14,15]. Kahn and colleagues have developed a pragmatic framework for data quality assessment in EHRs. Their framework assesses “fitness for use” in health care QI and comparative effectiveness research (CER) [7,13,16]. The National Institutes of Health (NIH) National Healthcare Research Systems Collaboratory has recently added a data quality review criterion for its research projects [17]. The National Academy of Medicine workshop confirmed that data quality is very important [9].

In 2010, several projects were funded by the Agency for Healthcare Research and Quality (AHRQ), afforded by the American Recovery and Reinvestment Act of 2009 [18]. The goal of this initiative was to aggregate data to both advance health system QI efforts and conduct CER. To achieve this goal, investigators leading Washington State’s Surgical Care and Outcomes Assessment Program (SCOAP) [19] participated in a funded project to establish the Comparative Effectiveness Research Translation Network (CERTAIN) [20]. SCOAP is a State approved Coordinated Quality Improvement Program [21] that operates under the non-profit Foundation for Health Care Quality [22]. SCOAP is an audit-and-feedback QI process led by clinicians. In Washington State, 55 of the state’s 60 hospitals participate by manually abstracting health record data about patients undergoing abdominal, oncologic, non-cardiac vascular, and spine surgical procedures, and entering these into the SCOAP registry database. Quarterly reports provide feedback about targeted benchmarks and reveal peer performance. This continuous QI loop has resulted in demonstrated improvements in the quality and safety of surgical care while decreasing costs [19].

CERTAIN built on SCOAP and established Washington State’s real-world learning health care system. The goal of CERTAIN’s inaugural project was to enhance the SCOAP QI registry by semi-automating data collection and validating these data, thereby creating a CDR for use in QI and CER. Unlike other multicenter data aggregation methods, this project did not require participating hospitals to normalize their data before delivery to the CDR. Instead, the project developed and tested a unique approach: all participating sites delivered raw EHR data, which was normalized at the CDR. Essentially, in an effort to unburden the cost of participation from individual sites, the project used a *centralized* federated model instead of a *distributed* federated model. We previously reported on the methods for our planned validation study [23]. This report describes the three phases of validation that were completed, and the performance of the automated abstraction system compared to the manual abstraction system and the EHR.

## Methods

### Settings

Our previous work describes in detail the participating hospitals, site engagements, data flow, and data element selection processes [23,24]. In brief, three of the four participating hospitals were part of a large, integrated academic health system in King County, Washington. The hospitals included two community hospitals, a tertiary care referral center, and a Level-I trauma center, with numbers of beds ranging from ~275 to ~450. Across the four hospitals the EHRs were Cerner, Siemens and two instances of Epic. Each hospital actively provided data to the SCOAP QI registry for 150 care processes that collectively represented 25 abdominal/oncology, non-cardiac vascular and spine surgical procedures. Each data collection form was composed of approximately 700 data elements, some common to all, others unique. (See example in Supplementary Figure 1.)

#### Validation Overview

Using a centralized federated data model that captured HL7 standard messages in real time, CERTAIN created a *site-specific CDR* for each participating site by installing the Amalga® platform from Microsoft (Redmond, WA, USA), now the Caradigm Intelligence Platform® from Caradigm (Seattle, WA, USA) [25]. The *CERTAIN site-specific CDR* retrieved data from all types of EHRs, storing message queues that permitted data manipulation independently from clinical systems. Virtual private networks (VPNs) allowed data to flow from select data streams (feeds) at each participating site to that site’s *CERTAIN site-specific CDR*, which was physically maintained at an off-site data center. Data were then standardized and normalized to a common data model, thereby creating the *CERTAIN combined CDR*.

Throughout the project (10/2012-7/2013), investigators operated manual and automated abstraction systems in parallel, and conducted three phases of validation. In phase 1, ingested data from 50 patients per site were validated. In phase 2, 20 randomly selected SCOAP cases per registry (abdominal, oncologic, non-cardiac vascular or spine), per site, were validated. In phase 3, matched pairs of transformed, automated data elements were compared to those manually abstracted. All discordant/valid pairs and 10 percent of concordant pairs were validated. In each phase, data elements (phases 1 and 2) or pairs of data elements (phase 3) were compared against the EHR (gold standard). Information retrieval statistics (precision, recall, F-score) were calculated. Iterative improvements were made until each matched the EHR with 95 percent accuracy. (Supplementary Figure 2.)

### Selection of Data Feeds and Data Elements

We undertook a two-step process to determine which EHR data sources to ingest and which SCOAP data elements to automate. First, CERTAIN investigators and SCOAP abstractors conducted an EHR audit to determine sources within each EHR from which SCOAP data elements were abstracted. We then compiled a short list of the most common EHR sources of SCOAP data. Second, from these common sources, investigators assigned an expected level of difficulty for automated abstraction and established a likely source feed for those considered abstractable. We describe these methods in our previous publication [26]. Specifically, we reviewed an aggregated spreadsheet of all data elements and labeled each as: 1) structured at all sites (e.g. age); 2) structured at some sites (e.g. lowest intraoperative body temperature; 3) machine-readable text (e.g. discharge diagnosis); or 4) too difficult to automate (e.g. no good electronic source or data contained in multiple sources). For example, in learning that abstractors used history and physical or pre-anesthesia dictation notes to determine smoking histories, investigators decided that smoking history could be retrieved only through the development and application of NLP algorithms.

These combined efforts resulted in identification of the top five data feeds across sites that would result in the maximum number of data elements abstracted to achieve automation: ADT (admissions, discharges, and transfers), laboratory services, dictations, radiology reports and medications/allergies. Supplementary Table 1 displays the feeds ultimately established based on each site’s ability to send a feed and the capability of the feed to ingest data.

Of over 700 data elements required on one or more SCOAP forms, we also initially estimated that the *theoretical maximum* proportion of elements that could feasibly be automated given unlimited time and resources was 10 percent for structured data and 38 percent for NLP. Abstraction of 21 percent of data elements would be difficult to abstract due to presence in multiple sources; no good source of electronic data was available for an additional 38 percent. The analysis further revealed that 37 percent of the abdominal/oncologic data form (reported together since abdominal and oncologic operations have overlapping core variables, but separate procedure targeted metrics), 28 percent of the non-cardiac vascular form, and 45 percent of the spine form could theoretically be automated. From the data sources *ultimately ingested*, up to 16 percent of the data elements from the abdominal/oncology SCOAP form could be abstracted (77 structured/45 NLP out of 740 possible data elements); 12 percent from the non-cardiac vascular form (36 structured/34 NLP out of 586); and 6 percent from the spine form (45 structured/7 NLP out of 882). Ultimately 29 percent of elements determined to be possible to automate were automated (244/833 elements). (See Table 1.)

**Table 1:** Amount of Data Feasibly Ingested.

	Abdominal/Oncologic n (%)	Non-cardiac Vascular n (%)	Spine n (%)

**Total number of data elements currently manually abstracted for SCOAP**	N = 740	N = 586	N = 882

Not possible to abstract	466 (63%)	422 (72%)	487 (55%)
Possible to abstract	274 (37%)	164 (28%)	395 (45%)
Structured	77 (10%)	36 (6%)	45 (5%)
NLP	45 (6%)	34 (6%)	7 (<1%)
Possible with additional resources	152 (21%)	94 (16%)	343 (39%)

**Total number elements actually automated in CERTAIN**	**122 (16%)**	**70 (12%)**	**52 (6%)**

**Of total number of data elements possible to abstract**	n = 274	n = 164	n = 395

Structured	77 (28%)	36 (22%)	45 (11%)
NLP	45 (16%)	34 (21%)	7 (2%)
Possible with additional resources	152 (56%)	94 (57%)	343 (87%)

**Total number elements actually automated in CERTAIN**	**122 (45%)**	**70 (43%)**	**52 (13%)**


NLP = natural language processing.

Finally, each data element targeted for abstraction was categorized as continuous, binary or categorical. An acceptable margin of error was set for continuous variables for use when comparing data elements from each data source (automated abstraction, manual abstraction, or EHR). For example, the margin of error for a glycosylated hemoglobin test was ±0.5 percent.

### Validation 1 – Data Ingestion

The task in validation 1 was to compare each data element from each feed, at each site, after it was extracted, transferred and loaded (ETL) into that site’s *CERTAIN site-specific CDR*, with the same data element in the EHR. This step took place before the data were transformed and normalized to the CERTAIN combined CDR data standard. The purpose was to determine the proportion of data elements that were correctly loaded from the EHR. For each site, feeds were tested with up to 50 patients. Patients were selected into the *CERTAIN site-specific CDR* based on hospital service description (patient underwent a SCOAP surgical procedure) and age ≥18 years. We ensured that each patient had completed their hospital stay (i.e. had been discharged) and that feeds were active throughout the hospital stay. All data displayed in the *CERTAIN site-specific CDR* were directly compared to the information displayed in the EHR. This was a reiterative process; as information was found to be displaying improperly, off-site CDR staff updated the extraction and parsing process, and the patient was revalidated. This process was conducted until the feeds accurately displayed data at least 95 percent of the time.

### Validation 2 – Comparing CERTAIN’s CDR automation of SCOAP data elements to the EHR

The task in validation 2 was to compare each data element contained in the CERTAIN combined CDR with the same data element in the EHR. The purpose was to determine the proportion of data elements in the combined CDR that were correctly transformed and normalized to the SCOAP format. For each site, up to 20 patients per SCOAP form were validated. As with validation 1, validation 2 was a reiterative process – as data elements were found to have been automated incorrectly, they were sent to off-site staff for review. These staff programmed a solution for each discrepancy and updated the parsers. The same patients were then revalidated for the same data element(s).

### Validation 3 – Comparing matched pairs of data elements for concordance, and then each with the EHR; calculation of information retrieval statistics

Three steps comprised this final validation phase. In the first step, SCOAP case records of transformed and normalized data elements abstracted through the CERTAIN combined CDR process were matched to and compared with the same data collected by manual abstraction. Because the CERTAIN combined CDR used only International Classification of Diseases-Clinical Modification-9^th^ Revision (ICD-9) and Clinical and Procedural Terminology (CPT) codes to populate a list of patients with SCOAP procedures, records from both sources were first matched based on medical record number or a combination of other patient identifiers, including date of birth, patient initials and surgery date. Matching was attempted for all abdominal/oncologic, non-cardiac vascular, and spine SCOAP cases present in either database.

In the second step, a random sample of 10 percent of matched cases was selected. Within each case, pairs of data elements (automated and manually abstracted) were matched, and the value from each source compared to the other. Each pair was classified into one of three categories: 1) Concordant/Valid: Values automatically abstracted and manually abstracted were exactly equal, or equal within the pre-specified margin of error; 2) Discordant/Valid: Values automatically abstracted and manually abstracted were not equal, yet both contained a response; 3) Discordant/Missing: A value was present from one system, but not the other. Pair concordance and discordance were calculated using percentages. Analysts were blinded as to the source of the values. All Discordant/Valid pairs were validated against the EHR (as the gold standard). We did not validate Discordant/Missing pairs as we focused on the concordance of data delivered, and relied on Validations 1 and 2 to provide complete data retrieval.

In the third step, using the values above, we calculated information retrieval metrics: precision, recall, and the F-score, which is the weighted harmonic mean of precision and recall that assesses the tradeoff between precision and recall. Precision is the fraction of retrieved documents that are relevant to the user’s information need and is the same as positive predictive value. Recall is the fraction of the relevant document in the collection that is retrieved, and is the same as sensitivity. These were calculated for pairs of manually abstracted and automatically abstracted data elements separately, comparing each to the EHR. To calculate each metric, we constructed a 2 × 2 table and used as cell counts the frequency of each data element from each source, in turn. We defined true positive as the data element being valid both by the specified abstraction method (manual or automated) and in the EHR. We defined false positive as the data element being valid by the specified abstraction method, but missing in the EHR. We calculated false negative as the data element being missing by the specified abstraction method, but valid in the EHR. We did not use a true negative category, as the data element would have been missing by both the specified abstraction method and in the EHR, leaving nothing to compare. (See Supplementary Table 2.)

We subsequently categorized metrics by section of the form (e.g. demographics). Next, using histograms, we graphed the distribution of each category of metric, thereby establishing cut-points for ease in interpreting. We defined a score of 0.8–1.0 as performing well; 0.5 to 0.8 performing satisfactorily; and <0.5 as performing poorly.

## Results

### Validation 1

Table 2 shows the final Validation 1 results by site. Not all feeds reached the 95 percent threshold due to time and resource constraints. For the medication/allergies feed, we report allergies only, as validation was not completed on medications due to the complexity of these multicomponent orders (e.g. generic name, strength, dose, dosage form).

**Table 2:** Results, Validation 1. Proportion of data elements that loaded correctly from each EHR, by feed.

Site	ADT	Labs	Dictations	Radiology Reports	Allergies

**A**	94%	98%	43%	N/A	N/A
**B**	89%	91%	Not completed	N/A	N/A
**C**	100%	100%	100%	100%	84%
**D**	99%	75%	99%	93%	85%

ADT = admission/discharge/transfer; Labs = laboratory services; N/A = Not available.

### Validation 2

Table 3 shows the Validation 2 results for each site, stratified by SCOAP form and grouped by feed, with SCOAP data elements contained within. The allergy portion of the medication/allergies feed was omitted from validation, as these data elements do not directly answer questions posed in the SCOAP forms. Across sites, forms, and feeds, the combined proportion of each data element that was correctly automatically abstracted and accurately represented SCOAP standards ranged from a low of 73 percent (site C, abdominal/oncologic form, laboratory feed) to 100 percent. The one exception to this was the laboratory feed for the vascular form at site D, where the proportion of data elements correctly represented was 34 percent. When data elements abstracted from structured data were combined with those for which abstraction required the use of NLP, the average proportion accurately represented increased to between 74 and 100 percent.

**Table 3:** Results, Validation 2. Proportions of data elements standardized, by site and SCOAP form.

Sites	SCOAP Form	Demographic %	Labs %	Medications %	Radiology Reports %	Dictations (NLP) %	Average %

**A**	Abdominal/Oncologic	97%	98%	90%	N/A	89%	94%
**A**	Vascular	92%	85%	77%	N/A		86%
**A**	Spine	94%	100%	93%	N/A		94%
**B**	Abdominal/Oncologic	97%	95%	97%	N/A	89%	95%
**B**	Vascular	98%	93%	97%	N/A		94%
**B**	Spine	94%	99%	98%	N/A		95%
**C**	Abdominal/Oncologic	99%	89%	N/A	94%	89%	93%
**C**	Vascular	96%	100%	N/A	N/A		95%
**C**	Spine	97%	90%	N/A	N/A		92%
**D**	Abdominal/Oncologic	99%	73%	N/A	89%	91%	88%
**D**	Vascular	98%	34%	N/A	N/A		74%
**D**	Spine	100%	89%	N/A	N/A		93%

Labs = laboratory; N/A = Not available; NLP = Natural language processing.

### Validation 3

Matches were found for 461 cases, comprising 32,906 data elements. Matching proportions for cases identified using manual versus automated methods varied from a low of 22 percent to a high of 76 percent; not all cases matched due to variations in how SCOAP patients are identified at different sites. Supplementary Table 3 shows the number of cases matched at each site by form.

We validated data elements for a generous random sample of over 10 percent (63) of matched cases. We oversampled to ensure we validated at least one form type from each site. Within this random sample, 3,068 pairs of data elements (average 80 percent) were concordant between abstraction methods. These proportions were similar by site, yet within each site, the proportions differed by type of SCOAP form. (See Supplementary Table 4.)

In the same 10 percent sample, 2,056 pairs of data elements were discordant. Within each pair, the results comparing the value from each abstraction method to the EHR appears in Table 4. In 5 percent of instances, (97 pairs) neither value correctly matched the EHR. In 58 percent of instances (1,185 pairs), manually abstracted values correctly matched the EHR, but automatically abstracted values did not. In 38 percent of instances (774 pairs), automatically abstracted values correctly matched the EHR, but manually abstracted pairs did not. In a few instances, values from both sources matched the EHR, but were discordant with each other. This may be explained by the fact that each value was equal to the EHR value within a pre-specified margin of error, but was not exact match within pair. For example, the margin of error for a blood glucose value was pre-specified as ±10 mg/dL. If the values were 110 mg/dL in the EHR, 117 mg/dL by automatic abstraction, and 105 mg/dL by manual abstraction, both the automatically abstracted and manually abstracted values match the EHR, as they are within the pre-specified margin of error, though they do not match each other. When discordance between pairs occurred, a greater proportion of manually abstracted than automatically abstracted values matched the EHR, but only by 20 percent (58 percent versus 38 percent), or 411 pairs (1,185–774). Stratifying these pairs in four dimensions; SCOAP form type; type of data element (binary, categorical or continuous); source of data (structured/NLP); and section of SCOAP form (e.g. demographics, risk factors) reveals variation in the rates of agreement among these categories.

**Table 4:** Results, Validation 3. Among Matched Cases, Number (%) of Discordant/Valid Pairs of Data Elements, Compared to EHR.

	Total N (%)	Neither Abstraction Method Matches EHR N (%)	Manually Abstracted Method Matches EHR N (%)	Automatically Abstracted Method Matches EHR N (%)	Both Methods Match EHR N (%)

**Pairs Reviewed**	2,056 (100%)	97 (5%)	1,185 (58%)	774 (38%)	2 <1%

**Hospital**					

A	757	30 (4%)	512 (68%)	215 (28%)	0
B	269	28 (10%)	157 (58%)	82 (30%)	2 (<1%)
C	294	14 (5%)	138 (47%)	142 (48%)	0
D	736	25 (3%)	336 (51%)	335 (46%)	0
**SCOAP Form**					

Abdominal/Oncologic	1,460	60 (4%)	828 (57%)	572 (39%)	0
Spine	350	22 (6%)	201 (57%)	126 (36%)	1 (<1%)
Vascular	246	15 (6%)	154 (63%)	76 (31%)	1 (<1%)
**Data Element Type**					

Binary	924	0	692 (75%)	232 (25%)	0
Categorical	776	60 (8%)	397 (51%)	319 (41%)	0
Continuous	356	37 (10%)	94 (26%)	223 (63%)	2 (1%)
**Source of Data**					

Structured	1,044	52 (5%)	396 (39%)	594 (57%)	0
NLP	1,012	45 (4%)	787 (78%)	180 (18%)	0
**Section of Form**					

Demographics	650	19 (3%)	236 (36%)	395 (61%)	0
Risk Factors	415	44 (12%)	264 (64%)	106 (25%)	1 (<1%)
Comorbidities	270	0	221 (82%)	49 (18%)	0
Pre-operative	45	1 (2%)	35 (78%)	9 (20%)	0
Intraoperative	199	21 (11%)	88 (44%)	89 (45%)	1 (<1%)
Perioperative	159	12 (8%)	107 (67%)	40 (25%)	0

Of the 3,068 concordant pairs evaluated, both values consistently matched the EHR 98 percent of the time for each hospital, SCOAP form, data element type, source of data, and section of the form. Ninety-five percent of categorical variables and 93 percent of risk variables matched, as it is difficult to find an exact match for each level of these. When discordant and concordant comparisons are combined, manual abstraction was accurate 92 percent of the time, and automated, 88 percent of the time. (Data not in table)

Statistical and rules-based NLP algorithms were created to abstract 27 data elements. Twenty-two (81.5 percent) of these performed with accuracy ≥90 percent. (See Supplementary Figure 3.)

When text mining accuracy was above 90 percent, automated abstraction performed as well as manual abstraction. Additional detail that describes the text mining algorithms can be found in our companion publication [27].

Results of the information retrieval statistics calculated on the 10 percent sample indicate that all demographic variables and the majority of perioperative processes, procedure characteristics and risk factors/comorbidities performed well. Laboratory values performed less well, and there was a trade-off between precision and recall for post-operative adverse events and interventions, albeit with only two variables. (See Table 5.)

**Table 5:** Results Information Retrieval Statistics.

Demographics/Admit/Discharge	Manual Abstraction			Automated Abstraction		

	Recall	Precision	F-score	Recall	Precision	F-score

# Variables	30	30	30	30	30	30
Score Category						
<0.5	0	0	0	0	0	0
≥0.5 & <0.8	0	0	0	0	0	0
≥0.8	100%	100%	100%	100%	100%	100%
**Laboratory Values**	**Manual Abstraction**			**Automated Abstraction**		

	**Recall**	**Precision**	**F-score**	**Recall**	**Precision**	**F-score**

# Variables	13	11	13	13	12	13
Score Category						
<0.5	23%	0	23%	8%	0	8%
≥0.5 & <0.8	46%	9%	23%	31%	0	0
≥0.8	31%	91%	54%	62%	100%	92%
**Perioperative Processes**	**Manual Abstraction**			**Automated Abstraction**		

	**Recall**	**Precision**	**F-score**	**Recall**	**Precision**	**F-score**

# Variables	27	26	27	27	26	27
Score Category						
<0.5	7%	0	4%	4%	0	4%
≥0.5 & <0.8	0%	0	4%	4%	0	4%
≥0.8	93%	100%	93%	93%	100%	93%
**Post-op Adverse Events /Interventions**	**Manual Abstraction**			**Automated Abstraction**		

	**Recall**	**Precision**	**F-score**	**Recall**	**Precision**	**F-score**

# Variables	2	2	2	2	1	2
Score Category						
<0.5	100	0	100	100	0	100
≥0.5 & <0.8	0	0	0	0	0	0
≥0.8	0	100	0	0	100	0
**Procedure Characteristics**	**Manual Abstraction**			**Automated Abstraction**		

	**Recall**	**Precision**	**F-score**	**Recall**	**Precision**	**F-score**

# Variables	73	71	73	73	61	73
Score Category						
<0.5	3%	0	3%	16%	0	16%
≥0.5 & <0.8	10%	1%	11%	8%	0	5%
≥0.8	88%	99%	86%	75%	100%	78%
**Risk Factor/Comorbidities**	**Manual Abstraction**			**Automated Abstraction**		

	**Recall**	**Precision**	**F-score**	**Recall**	**Precision**	**F-score**

# Variables	12	12	12	12	11	12
Score Category						
<0.5	8%	0	8%	17%	0	17%
≥0.5 & <0.8	0	0	0%	0	0	0
≥0.8	92%	100%	92%	83%	100%	83%

## Discussion

### Validation 1

The data abstraction endeavor was more complex than originally anticipated. There are several reasons why the proportion of data elements that correctly came through the feeds to the *CERTAIN site-specific CDRs* did not reach 100 percent. Fundamentally, there were problems with outbound data from each site. The manual abstraction system could overcome these; the automated system could not. First, SCOAP data abstractors are trained to find specific data elements and their temporal associations, and quickly learn workarounds to find missing data or correctly choose between multiple results. Second, some dictation documents were not appropriately labeled, so these were not abstracted when expected. Specifically, the range for dictations is wide because of inconsistent coding in the source system for site A, which led to error messages in the dictations viewer. Third, additional time was required to train the NLP algorithms to adjust for these differences among feeds. Fourth, data elements changed over time, both in the location within each EHR, and also when updates were made to SCOAP data collection forms. These reiterative processes required ongoing communication between off–site staff and each site to establish the feeds, and build and modify the parsers. Given sufficient time and resources, these issues could be addressed. Specifically, investing time to build an upfront infrastructure for abstracting individual data elements from each feed at each site and standardizing data across multiple sites would enable all feeds to function correctly.

### Validation 2

Validation 2 was also a lengthy and reiterative process. The solutions required of off-site staff varied over each of the four dimensions, feed, and site. Problems were identified with individual data elements. Importantly, a distinction must be made between static (date of birth) and dynamic (laboratory test results) data elements. Further, in many instances, the same piece of clinical data was found in different locations in the EHR. For example, a blood glucose laboratory test could appear as the result of a point-of-care test or of an intravenous laboratory draw. This was particularly evident for the SCOAP vascular form at Site D, where only 34 percent of automatically abstracted data elements were accurately represented. Finally, there was no longitudinal feature to data retrieval and interpretation. Thus, we were unable to establish temporal associations within or among data elements. Further, fewer data were available in structured format than was anticipated. Specifically, dictations were the source of more clinical data than originally thought. The NLP algorithms were necessary and useful in abstracting these; additional NLP algorithms could be developed to abstract even more data. Importantly, we were able to build a case list management tool that has the potential to be a great time saver for abstractors. With additional investments of time and personnel and evaluation of additional patients, the CERTAIN combined CDR has the potential to perform with greater validity.

### Validation 3

Fundamentally problematic was that we were unable to match all case records due to variations in methods of identification of SCOAP patients across sites. Further, there is much variability in these matching pairs created from the manual versus automated processes. Similar to what occurred in Validation 2, in many instances, discrepancies occurred because the same piece of clinical data was found in different locations in the EHR, sometimes at different time points, and was abstracted differently using either the manual or automated process. This was particularly true for laboratory values. Overall, a greater proportion of manually abstracted data elements matched the EHR when compared to the automated abstraction, but this difference amounted to only 411 pairs. A more overarching metric is that when discordant and concordant comparisons are combined, manual abstraction was accurate 92 percent of the time, and automated, 88 percent of the time. This pattern largely held for all sites and the four dimensions. The three exceptions for which automated abstraction performed better were continuous variables, structured data, and demographic variables. Moving forward, those implementing similar automated systems will achieve greater success by focusing on variables that perform well.

### Summary Discussion

Importantly, the data abstraction system worked as we would have expected. This was not the notable finding of the study. Rather, the notable finding was that data validation is critically important. Particularly impressive was the low proportion of data elements that exist within EHRs in structured format across different health care organizations. Also impressive was the sheer volume and variety of reasons that prevented us from achieving 100 percent accuracy with each validation metric.

Also, noteworthy is that of the 2,056 discordant/valid pairs in the 10 percent sample of cases, there is only a 20 percent difference between the proportion of manually abstracted elements that matched the EHR (58 percent) and the proportion of automatically abstracted elements that matched the EHR (38 percent). With manual abstraction requiring such intensive resources of time and personnel (and not being perfect either), investments in automated abstraction hold promise for conserving health care resources. Even so, our validation study reveals the nuances involved in validation and the complexities involved in preparing electronic data for use as real world evidence. It further points up that the current focus on data quality is on target.

Brown and Kahn have studied the issue of data quality extensively. They first called out five key concepts for use in the federated, albeit distributed, context: 1) attribute domain constraints; 2) relational integrity rules; 3) historical data rules; 4) state-dependent objects rules; and 5) attribute dependency rules [13]. *Attribute domain constraints* focus on anomalies in data values, (e.g. patients over the age of 150 years). *Relational integrity rules* compare data elements across data tables, (e.g. every patient who has a primary care visit should also appear in the demographic table). *Historical data rules* capture temporal relationships and trends. *State-dependent objects rules* extend temporal association rules to include logical consistency (e.g. diagnosis of breast cancer should precede treatment). *Attribute dependency rules* incorporate conditional dependence (e.g. only males should have a diagnosis of prostate cancer). Finally, they offer practical suggestions for assessing data quality in multisite networks. These include reviewing adherence to the common data model, reviewing each data domain, assessing expected clinical relationships, and assessing proprietary and human subjects concerns. More recently, these same investigators have conceptualized a “data lifecycle” that illustrates how data quality issues can result in data being ‘kicked back’ to an earlier point in the cycle. To remedy this challenge, they propose a data quality reporting framework comprised of 20 recommendations in four categories: Data Capture, Data Processing/Provenance, Data Elements/Characterization, and Analysis-Specific Documentation [7].

Our work provides a companion to that of Brown and Kahn in that we outline a series of steps for data quality checking in the context of creating a centralized federated model. Our model is unique in abstracting raw data from each disparate EHR, standardizing and normalizing it within the combined CDR. Yet, these lessons are specific neither to a centralized, federated model nor to the combined CDR system. The same challenges would apply to both a distributed federated model and projects involving other vendors, although to which entity the burden of the ETL process will fall differs among models. In a centralized model such as ours, the burden of the ETL process rests with the vendor. In a distributed model, such as the Sentinel Initiative [4], OHDSI Program [5], and PCORnet [6], the burden rests with each site that uploads data to the central repository. In theory, the centralized federated model such as ours offers greater opportunity to create a more complete dataset with burden placed largely on a single vendor rather than multiple sites with fewer resources, whereas with the distributed federated model, more limited amounts of data are loaded with burden on sites. Our work illustrates that careful crafting of the ETL methodology is critically important, and constantly checking its performance is only the first step. Once the CDR is populated, Kahn and Brown’s additional key concepts outlined for use by those using a distributed database must then be applied. Given additional resources (time and personnel), this analysis supports the future feasibility and functionality of automated EHR abstraction.

## Conclusion

In the learning health care system, users must be able to trust electronic clinical data for use as real world evidence to conduct QI initiatives and research. Our study was a grand experiment in that we used a unique approach to creating a CDR, specifically the use of a centralized federated model. To our knowledge, our project is the only one to have employed this approach, and our manuscript the only manuscript to describe the data quality checking and validation steps used therein. The major limitation of our project was that finite resources prevented us from continuing to improve data quality and establishing a fully automated data abstraction system. This limitation, however, is ultimately the strength of the study, as it pointed up the fact that although semi-automated data abstraction may eventually be useful, raw data collected as a byproduct of health care delivery is not immediately ready for use as real world evidence. More systematic approaches to gathering extant data and new approaches to analyzing these data are required to achieve the goal of the learning health care system.

**Detailed data reports for each step in the validation process for each site are available from the authors.

## Additional Files

The additional files for this article can be found as follows:

10.5334/egems.211.s1 Figure S1 Sample Surgical Care and Outcomes Assessment Program (SCOAP) Abdominal & Oncologic Data Collection Form.

10.5334/egems.211.s1 Figure S2 Validation – Overview of Data Flow.

10.5334/egems.211.s1 Figure S3 Results, Natural Language Processing Results.

10.5334/egems.211.s1 Table S1 Feeds Established.

10.5334/egems.211.s1 Table S2 2 × 2 table for calculating precision and recall.

10.5334/egems.211.s1 Table S3 Matched Case Records, by site and SCOAP form.

10.5334/egems.211.s1 Table S4 Among Matched Cases, Proportion of Concordant Pairs of Data Elements, Automated Compared to Manually Abstracted.
