# Supplementary material for: Automating Electronic Clinical Data Capture for Quality Improvement and Research: The CERTAIN Validation Project of Real World Evidence
Source: EGEMS (Wash DC). 2018 May 22;6(1):8. doi: 10.5334/egems.211 (PMC5983060; doi:10.5334/egems.211)
Supplement: Figure S1 — Sample Surgical Care and Outcomes Assessment Program (SCOAP) Abdominal & Oncologic Data Collection Form. [file egems-6-1-211-s1.pdf]

Supplementary Figure 1: Sample Surgical Care and Outcomes Assessment Program (SCOAP) Abdominal & Oncologic Data Collection Form

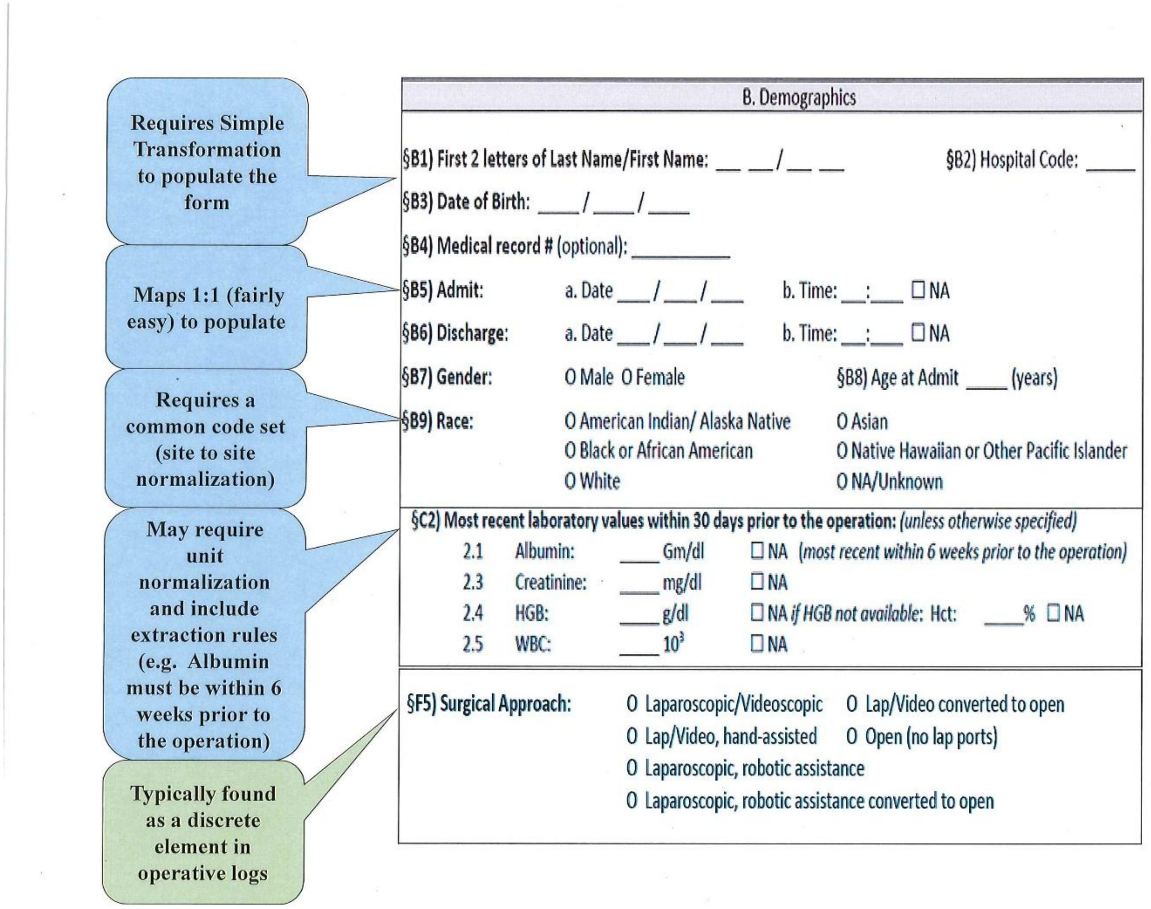

Supplementary Figure 2: Validation - Overview of Data Flow

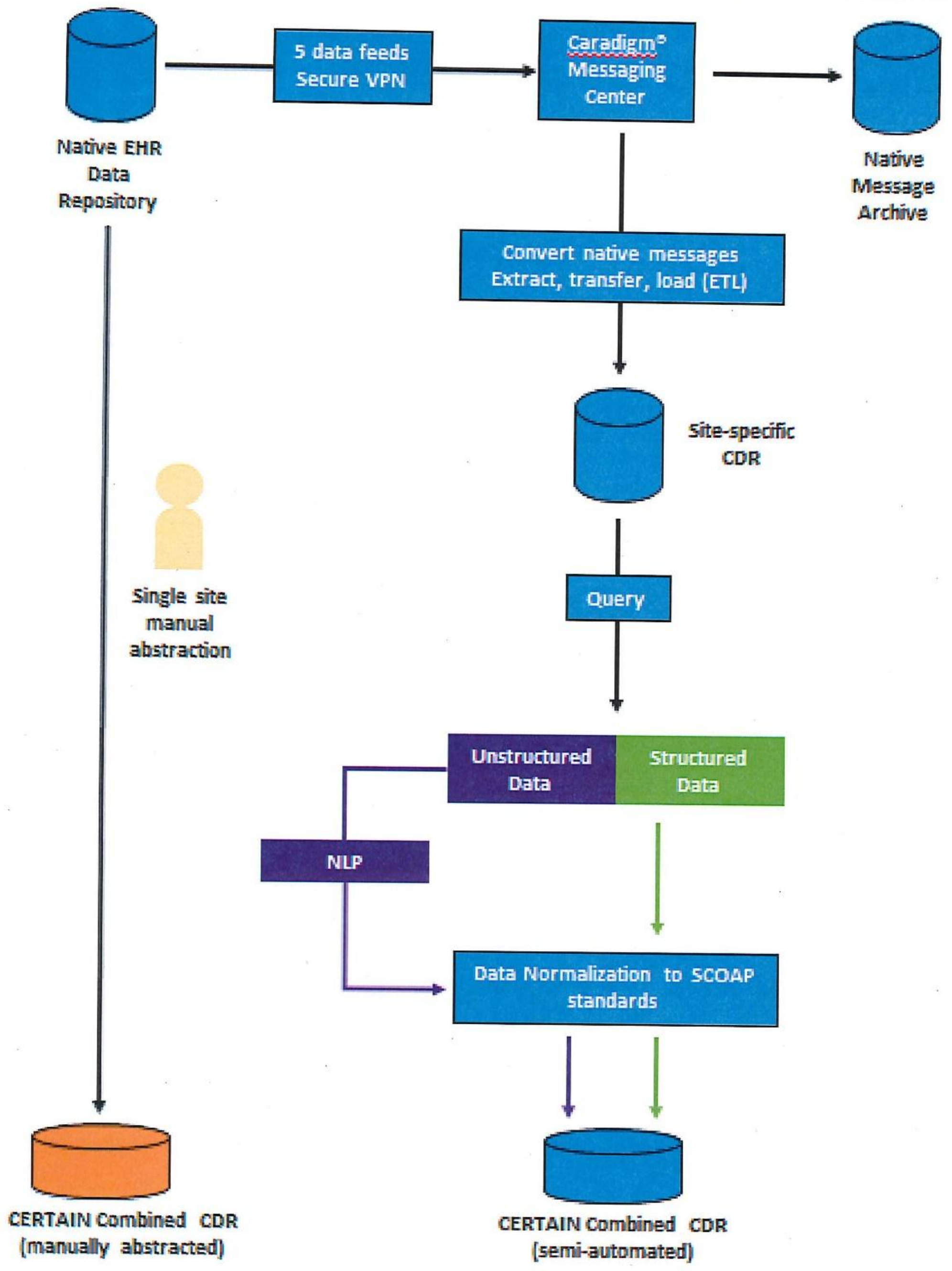

CDR = clinical data repository; CERTAIN = Comparative Effectiveness Research Translation Network; NLP = natural language processing; VPN = virtual private network

Supplementary Figure 3: Results, Natural Language Processing Results

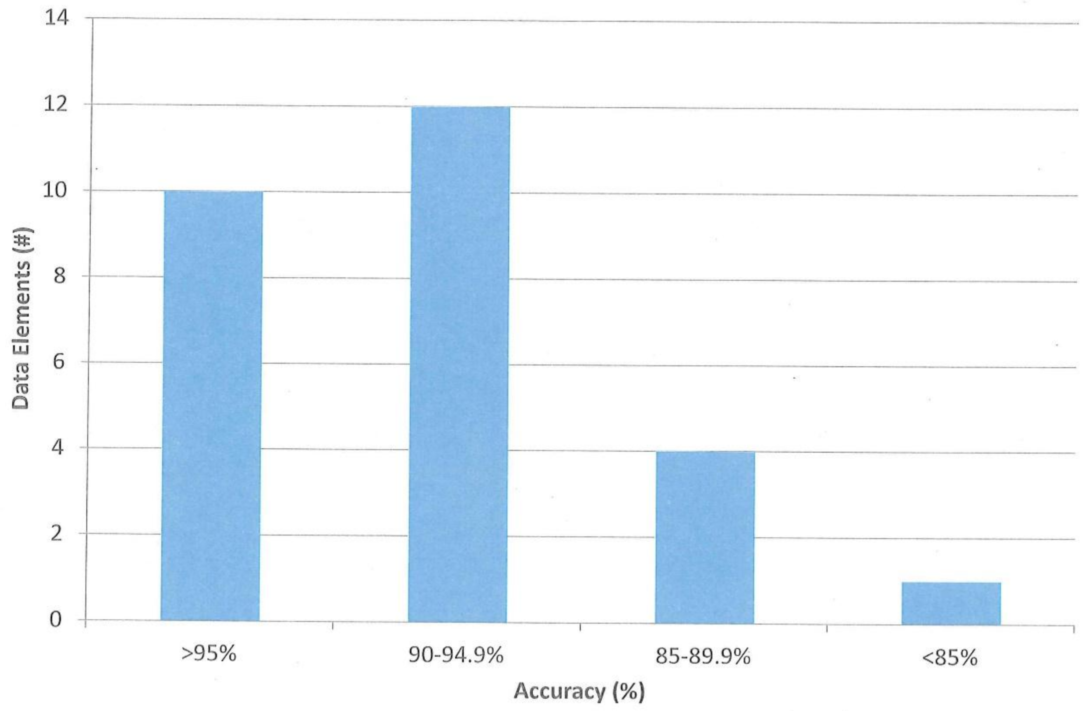

Supplementary Table 1: Feeds Established

Site	ADT	Labs	Dictations	Radiology Reports	Medications/ Allergies
A	yes	yes	yes	no	yes/no
B	yes	yes	yes	no	yes/no
C	yes	yes	yes	yes	yes/yes
D	yes	yes	yes	yes	no/yes

ADT = admission/discharge/transfer; Labs = laboratory services

Supplementary Table 2: 2 x 2 table for calculating precision and recall

	**EHR**	**EHR**	
Abstraction Method	TP:	FP:	Precision = PPV: TP/(TP+FP)

	V_{AM} and V_{EHR}	V_{AM} and M_{EHR}	
Abstraction Method	FN:<br>M_{AM} and V_{EHR}	TN:<br>M_{AM} and M_{EHR}	
	Recall = Sensitivity: TP/(TP+FN)		

Abstraction Method (AM) was either manual or automated; EHR = electronic health record FN = false negative; FP = false positive; M = missing; PPV = Positive predictive value; TN = true negative TP = true positive; V = valid; Unable to calculate true negative

Supplementary Table 3: Matched Case Records, by site and SCOAP form

**Case Records**	**Frequency (%)**				**Total Number Matched**
**Abdominal/ Oncologic**	**A**	**B**	**C**	**D**	
Manual*	42 (18%)	7 (19%)	7 (11%)	11 (7%)	
Automated*	64 (27%)	16 (44%)	11 (17%)	27 (17%)	
Matched	131 (55%)	13 (36%)	47 (72%)	118 (76%)	309
**Total**	**237 (100%)**	**36 (100%)**	**65 (100%)**	**156 (100%)**	
**Vascular**					
Manual*	5 (33%)	3 (9%)	10 (33%)	1 (5%)	
Automated*	5 (33%)	13 (37%)	5 (17%)	7 (33%)	
Matched	5 (33%)	19 (54%)	15 (50%)	13 (62%)	52
**Total**	**15 (100%)**	**35 (100%)**	**30 (100%)**	**21 (100%)**	
**Spine**					
Manual*	10 (12%)	57 (52%)	NA	63 (55%)	
Automated*	30 (36%)	29 (26%)	NA	18 (16%)	
Matched	43 (52%)	24 (22%)	NA	33 (29%)	100
**Total**	**83 (100%)**	**110 (100%)**	**NA**	**114 (100%)**	

**Overall Total**	**179 (47%)**	**56 (37%)**	**62 (61%)**	**164 (56%)**	**461**

*unmatched

Supplementary Table 4: Among Matched Cases, Proportion of Concordant Pairs of Data Elements, Automated Compared to Manually Abstracted

**Site**	**Pair Type**	**Type of SCOAP Form**			
		**Abdominal/ Oncologic**	**Vascular**	**Spine**	**Average**
A	Concordant	77%	72%	87%	79%
B	Concordant	70%	89%	86%	82%
C	Concordant	76%	69%	-	73%
D	Concordant	77%	81%	87%	82%

*Discordant pairs not shown as they are the complement of concordant pairs.
